# Prophylactic versus reactive leech therapy for venous congestion after fingertip replantation: A retrospective comparative study and literature review

**DOI:** 10.1016/j.jpra.2025.09.009

**Published:** 2025-09-12

**Authors:** Yusuke Kameda, Makoto Motomiya, Naoya Watanabe, Mitsutoshi Ota, Norimasa Iwasaki

**Affiliations:** a Department of Orthopaedic Surgery, Hokkaido Orthopaedic Memorial Hospital, Sapporo, Japan; b Department of Orthopaedic Surgery, Obihiro Kosei Hospital Hand Centre, Obihiro, Japan; c Hokkaido University, Department of Orthopaedic Surgery, Faculty of Medicine and Graduate School of Medicine, Sapporo, Japan

**Keywords:** *Hirudo medicinalis*, Finger, Replantation, Venous insufficiency, Bloodletting

## Abstract

**Introduction:**

Although the efficacy of medicinal leech therapy for managing venous congestion after fingertip replantation is well recognized, an optimal, standardized application protocol remains undefined. This study evaluated clinical outcomes under two application strategies and conducted a literature review to explore optimal protocols.

**Materials and methods:**

We retrospectively analyzed 25 digits that received leech therapy after fingertip replantation or revascularization at our institution (April 2015–March 2023). Group A (*n* = 12) underwent reactive leeching in response to clinical congestion, whereas Group B (*n* = 13) received prophylactic leeching two to three times daily from the early postoperative period, prior to any clinical signs of congestion. A literature search was conducted in accordance with PRISMA 2020 guidelines.

**Results:**

Complete survival was achieved in 8/12 digits (67 %) in Group A and 12/13 (92 %) in Group B. Early postoperative complications occurred in 6/12 versus 1/13 digits, and re-operations in 6/12 versus 1/13 digits, both significantly more frequent in Group A (*p* = 0.030). Only one Group A patient required transfusion owing to preexisting anemia. One case of *Aeromonas hydrophila* infection was identified. Functional and aesthetic outcomes were satisfactory in both groups. Combining our data with 129 digits from the literature yielded an overall survival rate of 81 % (124/154), although few reports detailed specific leech protocols.

**Conclusions:**

Effective bleeding control and timely congestion prevention are essential in artery-only fingertip replantation. A prophylactic, regularly scheduled leeching protocol appears to improve outcomes and may be adopted as a standard approach given its favorable risk-benefit profile.

## Introduction

Fingertip replantation offers substantial functional and aesthetic benefits.^1^^,^^2^ However, post-operative venous congestion remains a significant challenge. Venous anastomosis is recommended to improve survival,^3^ yet it is technically demanding because fingertip veins are minute and often crushed.^4^ Even with successful repair, venous outflow may be insufficient, requiring additional drainage.^3^^,^^4^

Various techniques have been proposed to manage congestion, including external bloodletting, arteriovenous anastomosis,^5^ and delayed venous drainage.^6^ Among these, external bloodletting is widely used for its simplicity and low technical demands. Reported methods include serial stab incisions, mechanical scrubbing, nail plate removal,^7^^,^^8^ local subcutaneous injection of heparin “(chemical leech),”^9^ and the use of medicinal leeches.^10^ Nonetheless, stable and adequate bleeding is difficult to maintain, and no standard protocol exists.

Medicinal leeches exert unique physiological effects through secreted substances like hirudin, histamine-like products, and hyaluronidase, which enhance bleeding and microcirculation.^11^ Despite these potential benefits, few studies have evaluated its clinical efficacy or optimized timing and frequency, and no standardized protocol exists. Moreover, complications such as transfusion need,^12^ *Aeromonas hydrophila* infection,^10^ and psychological distress for the patient^13^ have been reported, but not thoroughly studied.

This study aimed to compare two leech application protocols for fingertip replantation and review the literature to support the development of a standardized approach.

## Materials and methods

This retrospective study was approved by our ethics committee (number: 2023–017). We reviewed 60 patients (66 digits) who underwent digital replantation or revascularization distal to the distal interphalangeal (DIP) or thumb interphalangeal joint by four hand surgeons between April 2015 and March 2023. After excluding cases with failed revascularization (*n* = 8), no bloodletting (*n* = 30), or bloodletting without leeches (*n* = 3), 25 digits in 23 patients were included.

Collected variables included demographics, amputation characteristics, operative/bloodletting details, survival, complications (Clavien-Dindo classification),^14^ and clinical outcomes. Replantation was defined as complete amputation; cases with preserved tendon or nerve were classified as revascularization. Amputation level (Tamai, Ishikawa),^15^^,^^16^ injury mechanism (sharp, blunt, crush, avulsion) were recorded.^17^, ^18^, ^19^

Bloodletting parameters included timing, frequency, duration of leech therapy, and haemoglobin decline. Clinical outcomes included sensory recovery (Semmes–Weinstein [S–W] test), pulp atrophy, nail deformity,^20^ pain (visual analogue scale [VAS] score), cold intolerance, DIP joint motion, grip strength, the Fingertip Injuries Outcome Score (FIOS),^21^ and Disabilities of the Arm, Shoulder and Hand (DASH) score.

Pulp atrophy was graded based on final follow-up photographs as the fingertip width percentage of normal: normal (≥90 %), slight (80–89 %), moderate (70–79 %), severe (<70 %). FIOS was classified as excellent (≤12), good (13–18), fair (19–24), or poor (>24) per Jerome et al.^21^

### Surgical procedure and postoperative anticoagulation protocol

Devitalized and contaminated tissue was debrided under microscopy. Arteries were identified and tagged. Bony fixation was performed with Kirschner wires, with soft steel wires added as needed. One or two arterial anastomoses were performed, and venous anastomoses were attempted at the surgeon’s discretion.

Postoperatively, heparin was adjusted to maintain an activated partial thromboplastin time (aPTT) of 40–50 s. Dextran (500 mL/day), urokinase (240,000 U/day on days 0–3 and 120,000 U/day on days 4–7), and prostaglandin E1 (80 µg/day on days 0–3 and 40 µg/day on days 4–7) were also administered.

### Bloodletting protocol

Informed consent was obtained before initiating leech therapy. Two protocols were applied depending on the treatment period.

## Group A (Reactive Leeching Group)

Immediately after surgery, a stab incision was made at the fingertip, covered with gauze soaked in heparin (1000 U/mL), and scrubbed hourly. Circulation was assessed by bleeding characteristics and Doppler signals. Leeches were applied upon worsening signs of venous congestion (e.g., dark bleeding, diminished Doppler signals). After detachment, heparinized gauze was reapplied. Recurrent congestion prompted additional leech applications.

## Group B (Prophylactic Leeching Group)

**Figure 1:**
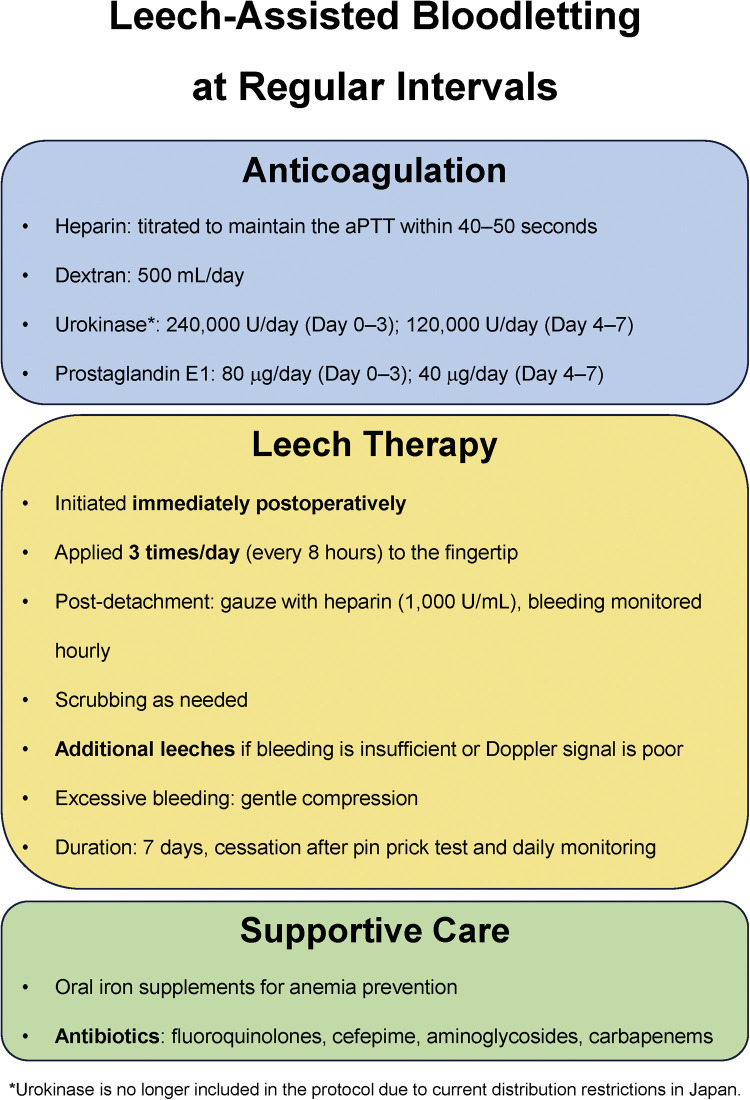
Institutional protocol for prophylactic leech therapy.

Due to necrosis cases under the Group A protocol, a revised regimen was introduced in 2019. Leeches were applied prophylactically two to three times daily from the early postoperative period, before signs of congestion. Initially, leeches were applied every 12 h following Brody et al.,^22^ but the interval was later shortened to every 8 h due to inadequate bleeding in some cases. Additional leeches were used when bleeding was insufficient or arterial signals declined, regardless of schedule (Figure 1).

In both groups, excessive bleeding was managed with light compression. Bloodletting continued for approximately 7 days. Circulation was carefully reassessed on the final day of therapy, and recurrent congestion was excluded using a pinprick test. Oral iron was supplemented as needed, and antibiotics were prescribed at the surgeon’s discretion to prevent *Aeromonas* infection.

### Statistical analysis

Continuous variables were expressed as mean ± standard deviation (for age) or median with interquartile range (IQR), as appropriate. Group comparisons were performed using the chi-squared test, Fisher’s exact test, unpaired *t*-test, or Mann–Whitney *U* test, depending on data distribution. Statistical analyses were conducted using GraphPad Prism (version 10.4.2; GraphPad Software, Boston, MA, USA), with *p* < 0.05 considered statistically significant.

### Literature search method and study selection criteria

A PRISMA-guided literature search was conducted on March 25, 2025, using PubMed, Web of Science, and EBSCOhost with keywords combining “finger replantation” or “digital replantation” with “leech,” “venous congestion,” or “artery only.”^23^ Studies were included if medicinal leeches were used to treat venous congestion following fingertip replantation or revascularization.

Exclusion criteria were as follows: non-fingertip amputations, studies combining leech and non-leech cases without subgroup analysis, absence of survival outcome data, non-English language articles, review articles without case data, and irrelevant topics.

**Figure 2:**
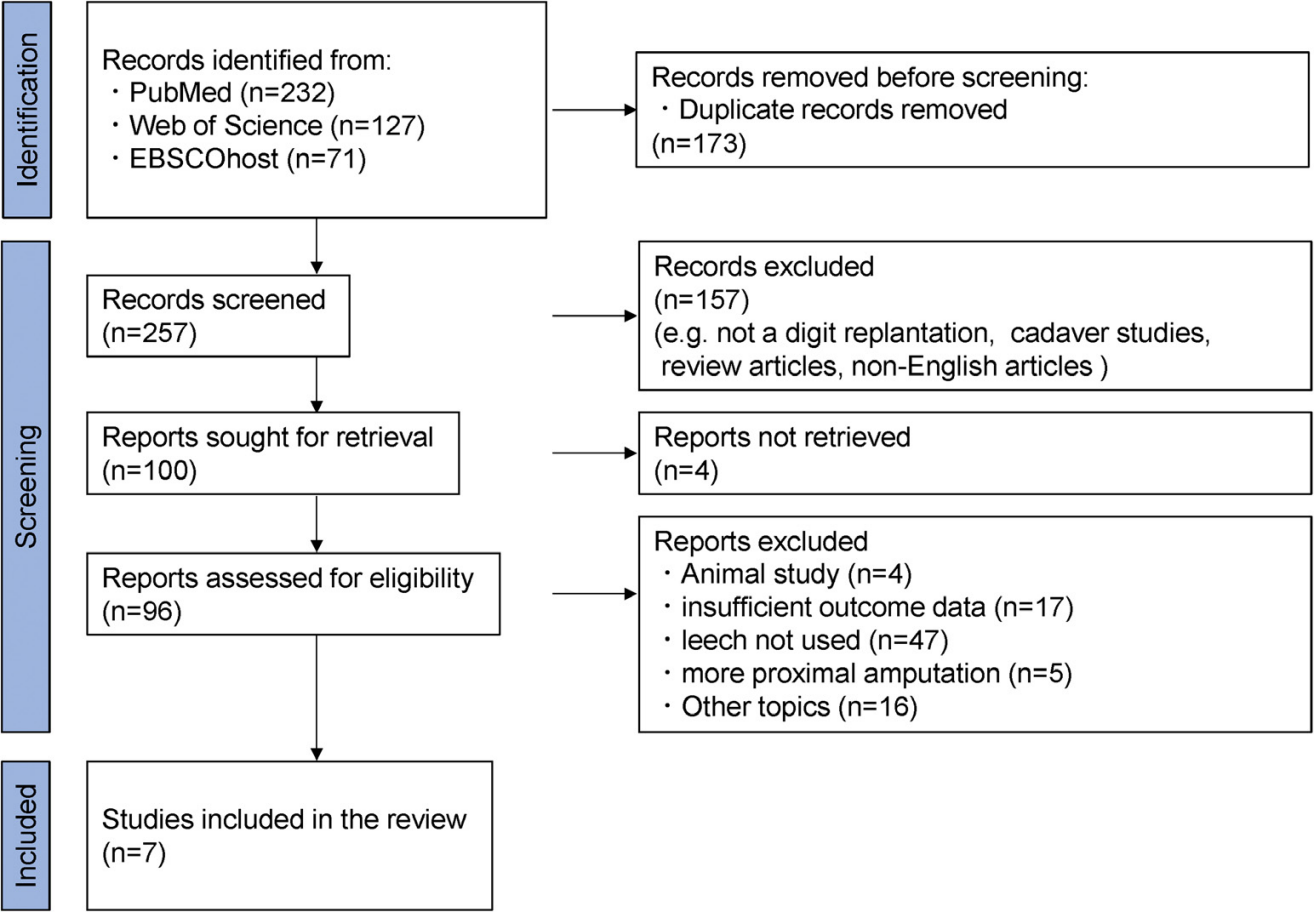
PRISMA 2020 flow diagram.

The study selection process is illustrated in the PRISMA flow diagram (Figure 2).

### Data extraction and synthesis

Two independent reviewers initially screened the titles and abstracts for eligibility. Full texts of potentially eligible studies were collected, and data on study design, amputation level, initiation timing, frequency and duration of leech therapy, survival rate, complications were extracted. Data analysis was performed using only the available data. Case reports were qualitatively assessed using the National Institutes of Health evaluation tool.^24^

## Results

### Patient demographics and amputation characteristics

Table 1, Table 2 summarize patient demographics and amputation characteristics. A total of 25 digits from 23 patients (17 men [19 digits] and six women [6 digits]; mean age 45 ± 13 years) were included. Replantation was performed in 23 digits and revascularization in two. Injured digits included one thumb, eight index, six middle, eight ring, and two little fingers. Injury mechanisms were blunt (*n* = 16), crush (*n* = 6), and sharp (*n* = 3). According to the Ishikawa classification, amputation levels were subzone 2 (*n* = 10), subzone 3 (*n* = 13), and subzone 4 (*n* = 2). Group A included 12 digits and Group B 13, with no significant baseline differences.

**Table 1:** Patient demographics.

		Total	Group A	Group B	*p* value
Number of patients/digits		23/25	12/12	11/13	
Age		45 ± 13	42 ± 15	48 ± 11	0.251
Sex	Male Female	19 6	8 4	11 2	0.378
Comorbidity			3	5	0.673
	DM		2	0	0.220
	HTN		2	1	0.593
	Others		1	5	0.160
Smoking		11	5	6	>0.99
Use of antithrombogenic agents		0	0	0	>0.99

DM, diabetes mellitus; HTN, hypertension.

Continuous variables were expressed using the mean ± SD.

**Table 2:** Amputation characteristics.

	Total	Group A	Group B	*p* value
Number of digits	25	12	13	
Replantation/Revascularization	23/2	11/1	12/1	>0.99
Finger				0.662
Thumb	1	0	1	
Index	8	4	4	
Middle	6	3	3	
Ring	8	5	3	
Little	2	0	2	
Type of injury				0.720
Avulsion	0	0	0	
Crush	6	2	4	
Blunt	16	8	8	
Sharp	3	2	1	
Subzone (Ishikawa)				0.369
1	0	0	0	
2	10	5	5	
3	13	5	8	
4	2	2	0	

### Operative details

Table 3 summarizes the operative details. A single arterial anastomosis was performed in 24 digits, whereas one digit in Group B underwent two anastomoses. Venous anastomoses were attempted in 10 digits, but all were unsuccessful, necessitating bloodletting due to inadequate venous return. Operative time per digit was comparable between groups. However, digits in which venous anastomoses were attempted (*n* = 8) had significantly longer operative times than those without (*n* = 13) (median: 389 min [IQR: 276–545] vs 168 min [IQR: 157–326]; *p* = 0.011).

**Table 3:** Operative details.

	Total	Group A	Group B	*p* value
Number of patients/digits	23/25	12/12	11/13	
Number of artery anastomoses (vein grafts used)				>0.99
1	24 (3)	12 (2)	12 (1)	
2	1	0	1	
Digits with attempted venous anastomosis (vein grafts used)	10 (7)	6 (3)	4 (4)	0.428
Blood loss during surgery (ml)	0 (0–0)	0 (0–38)	0 (0–0)	0.917
Operation time per digit (min)^a^	293 (168–384)	293 (250–392)	260 (167–370)	0.394

Continuous variables were expressed using the median (interquartile range).

a Four digits excluded due to inability to accurately measure operative time as a result of concurrent treatment for associated injuries at other sites.

### Details of medicinal leech application for bloodletting

Leech-related data are summarized in Table 4. Bloodletting duration was comparable between groups (median: 8 days), but the number of leeches used per digit was significantly higher in Group B (median: 17) compared with Group A (median: 5). Leech application was initiated significantly earlier in Group B (all cases on postoperative day 0) compared with Group A (median: postoperative day 1). Four digits in Group B required unscheduled application within 8 h due to insufficient early bleeding. One digit in Group A developed excessive bleeding, managed with brief compression.

**Table 4:** Details of bloodletting.

	Total	Group A	Group B	*p* value
Number of patients/digits	23/25	12/12	11/13	
Bloodletting duration (days)	8 (7–10)	8 (6–12)	8 (7–9)	0.595
Number of leeches per digit	13 (5–17)	5 (2–9)	17 (16–24)	<*0.001*⁎⁎⁎
Time to initiation of leech (days)	0 (0–1)	1 (0.3–3.5)	0 (0–0)	*0.013*⁎
Leeching at <8-h intervals	4	–	4	
Peak aPTT during bloodletting (s)	44.5 (39.1–61.2)	42.3 (40.7–58)	48.4 (36.8–63.3)	0.351
Bleeding requiring compression	1	1	0	0.480
Iron supplementation	13	6	7	0.680
Hb decrease during bloodletting (g/dl)	2.5 (1.7–3.8)	2.2 (1.2–3.5)	2.5 (1.7–4.0)	0.466
Patients receiving antibiotics	23	12	11	
Resistant to *Aeromonas*	10 (CEZ 10)	5 (CEZ 5)	5 (CEZ 5)	>0.99
Sensitive to *Aeromonas*	15(GM13, PIPC/TAZ 1, CFPM 1)	7 (GM 6, PIPC/TAZ 1)	8 (GM 7, CFPM 1)	

aPTT, activated partial thromboplastin time; Hb, Haemoglobin; CEZ, cefazolin; GM, gentamicin; PIPC/TAZ, piperacillin/tazobactam; CFPM, cefepime.

Continuous variables were expressed using the median (interquartile range).

⁎ *p* < 0.05.

⁎⁎⁎ *p* < 0.001.

Haemoglobin reduction was comparable (Group A: 2.2 g/dL; Group B: 2.5 g/dL). Antibiotics effective against *Aeromonas* were used in 15 digits; cefazolin alone was used in the remaining 10. No patients discontinued therapy due to psychological distress.

### Survival rate and complications

Table 5 shows complete survival in 8/12 digits (67 %) in Group A and 12/13 (92 %) in Group B (*p* = 0.062). Complete necrosis occurred in three Group A digits (subzone 3) on postoperative days 5–8; two were terminalized, and one was covered with a thenar flap. Partial necrosis developed in one digit in each group, both complicated by deep infection and managed with debridement: methicillin-resistant coagulase-negative staphylococci in Group A and *Aeromonas hydrophila* in Group B (details in Table 6).

**Table 5:** Survival rate and complications.

	Total	Group A	Group B	*p* value
Number of patients/digits	23/25	12/12	11/13	
Complete survival	20	8 (67 %)	12 (92 %)	0.062
Postoperative complications				
No complications	16	5	11	*0.041*⁎
Grade 1	0	0	0	
Grade 2	1	1	0	
Grade 3a	8	6	2	
Grade 3b	1	1	0	
Early complications (<3 weeks)	7	6	1	0.030*
Total necrosis	3	3	0	0.096
Partial necrosis	2	1	1	>0.99
Arterial spasm	1	1	0	0.480
Blood transfusion	1	1	0	0.480
Postoperative infection	3	2 (MRSA 1, MRCNS 1)	1 (*Aeromonas hydrophila*)	0.593
Delayed complications (>3 weeks)	3	2	1	0.593
Delayed osteomyelitis	1	0	1 (MSSA)	>0.99
Nonunion	1	1	0	0.480
Tendon adhesion	1	1	0	0.480
Additional surgery until wound healing	7	6	1	*0.030*⁎
Terminalization	2	2	0	0.220
Arterial reanastomosis	1	1	0	0.480
Local flap	2	2	0	0.220
Debridement	3	2	1	0.593
Additional surgery after wound healing	3	2	1	0.593
Tenolysis	1	1	0	0.480
DIP arthrodesis	1	1	0	0.480
Debridement	1	0	1	>0.99

MRSA, Methicillin-Resistant *Staphylococcus aureus*; MRCNS, Methicillin-Resistant Coagulase-Negative Staphylococci; MSSA, Methicillin-Sensitive *Staphylococcus aureus*.

⁎ *p* < 0.05.

**Table 6:** Cases of complete and partial necrosis.

Age/Sex	Group	Subzone (Ishikawa)	Bloodletting duration (days)	Number of leeches	Time to initiation of leech therapy (postoperative day)	Complication	Additional surgery	FIOS	DASH score
18/F	A	3	4	6	Day 1	Complete necrosis (POD5)	Local flap	16 (Good)	4.5
32/M	A	3	6	2	Day 4	Complete necrosis (POD6)	Amputation	20 (Fair)	11.4
48/M	A	3	8	10	Day 0 (after congestion onset)	Complete necrosis (POD8)	Amputation	23 (Fair)	11.4
44/M	A	2	17	17	Day 0 (after congestion onset)	Partial necrosis Osteomyelitis (MRCNS) Nonunion	Debridement Local flap	17 (Good)	9.1
43/M	B	3	14	31	Day 0 (before congestion onset)	Partial necrosis	Debridement	19 (Fair)	4.5

FIOS, Fingertip Injuries Outcome Score; POD, postoperative day; MRCNS, Methicillin-Resistant Coagulase-Negative Staphylococci.

Early complications and reoperations were significantly more common in Group A. One anaemic patient in Group A required transfusion (6 units). One superficial infection caused by methicillin-resistant *Staphylococcus aureus* was observed in Group A and managed with debridement. Early arterial spasm occurred in one digit in Group A on postoperative day 2. The vessel recovered with topical application of papaverine alone, and an additional arterial anastomosis was performed as a safety measure.

Late events were comparable. Group B had one delayed methicillin-sensitive *Staphylococcus aureus* osteomyelitis following Kirschner-wire exposure; Group A had an asymptomatic nonunion and one tenolysis for tendon adhesion.

## Clinical outcomes

Clinical outcomes of completely surviving digits are summarized in Table 7. Protective sensation (S-*W* ≤ 4.31) returned in 15/18 digits (83 %). Moderate-to-severe pulp atrophy occurred in 2/20 digits (10 %), and moderate-to-severe nail deformity in 11/20 digits (55 %). Residual pain was noted in 6 digits. The median FIOS and DASH scores were 15 and 2, respectively.

**Table 7:** Clinical outcomes of completely surviving digits.

	Total	Group A	Group B	*p* value
Number of survived digits	20/25	8/12	12/13	
Follow up (days)	351 (247–875)	260 (140–660)	370 (302–875)	0.240
S-W test^a^				0.596
2.83	2	0	2	
3.61	8	5	3	
4.31	5	0	5	
4.56	2	1	1	
>6.65	1	1	0	
Pulp atrophy				0.785
None	11	4	7	
Slight	7	4	3	
Moderate	2	0	2	
Severe	0	0	0	
Nail deformity				0.858
None	4	0	4	
Slight	5	4	1	
Moderate	7	4	3	
Severe	4	0	4	
Pain (VAS score)	0 (0–15)	2.5 (0–39)	0 (0–0)	0.302
Cold intolerance	11	6	5	0.197
Range of motion (DIP arc)^b^	49 (32–66)	66 (27–72)	44 (32–60)	0.675
Grip strength (kg)	34 (21–38)	22 (16–37)	37 (31–41)	0.071
Grip strength (% of contralateral side)	86 (75–92)	78 (63–91)	88 (77–98)	0.189
FIOS	15 (12–16)	16 (13–18)	15 (12–16)	0.251
Excellent	6	2	4	
Good	12	4	8	
Fair	3	2	0	
Poor	0	0	0	
DASH score	2 (2–9)	9 (2–9)	2 (1–10)	0.537

^a^Excluding 2 cases without measurements.

^b^Excluding 4 digits (DIP arthrodesis).

Continuous variables were expressed using the median (interquartile range).

No significant differences in clinical outcomes were found between groups. However, digits with necrosis had a significantly worse median FIOS than those with complete survival (19 vs. 15; *p* = 0.017).

### Review results

A comprehensive review identified seven case series on medicinal leech therapy for bloodletting; no comparative studies were found.^4^^,^^12^^,^^22^^,^^25^, ^26^, ^27^, ^28^ Including 25 digits from the present study, 154 digits were analyzed for amputation characteristics and outcomes (Table 8). Amputation levels were Tamai zone 1 in 91 digits, zone 2 in 45, and unclassified in 18. The overall survival rate was 124/154 digits (81 %), with 78/91 (86 %) in zone 1 and 38/45 (84 %) in zone 2.

**Table 8:** Summary of previous reports on leech therapy following fingertip replantation.

Author	Zone 1 survival	Zone 2 survival	Leech initiation^a^	Leech frequency	Bloodletting duration (days)	Systemic anticoagulation	Complication	Quality assessment score
Brody et al.^22^	–	100 % (3/3)	Prophylactic	Every 12 h	3–6	Aspirin + Dextran	No transfusion No infection	5/9
Foucher et al.^27^	64 % (7/11)	73 % (8/11)	–	–	5	–	–	4/9
Akyürek et al.^25^	67 % (10/15)	–	–	–	10–12	Aspirin + Heparin + Dextran	–	5/9
Breahna et al.^26^	Zone 1 + 2: 44 % (8/18)^b^		–	–	–	–	–	5/9
Ryu et al.^4^	93 % (41/44)	–	Reactive	–	–	PGE1 + Heparin	–	5/9
Hirase et al.^28^	100 % (7/7)	100 % (1/1)	Reactive	–	–	Dextran + Urokinase	–	5/9
Buntic et al.^12^	100 % (4/4)	100 % (15/15)	Reactive	–	6	Aspirin + Heparin + Dextran	Transfusion: 65 % (11/17)	6/9
*Our study*	90 % (9/10)	73 % (11/15)	Reactive (*n* = 12) Prophylactic (*n* = 13)	Occasional (*n* = 12) Every 8 h (*n* = 13)	5–18	PGE1 + Heparin + Dextran + Urokinase	Transfusion: 4 % (1/23 patients) Infection: 16 % (4/25 digits)	8/9

–: indicates data not reported in the original article.

^a^Categorized as “Prophylactic” (before congestion onset) and “Reactive” (at or after congestion onset).

^b^Since Zones 1 and 2 were not separately identified, the survival rate was presented as a combined total.

Systemic anticoagulation was reported in all 114 digits with available data. Leech application methods were described in 99 digits, though rarely in detail. Among these, 83 digits received leeches after the onset of venous congestion, resulting in a survival rate of 76/83 digits (92 %), while 16 digits received leech therapy prior to the onset of congestion, with 15/16 digits (94 %) surviving.

Only a few studies reported application frequency. Duration of therapy ranged from 3 to 18 days. Blood transfusions were required in 12/43 patients (28 %), and infections occurred in 4/28 digits (14 %).

## Discussion

### Effectiveness of a standardized leech application protocol

Although medicinal leech therapy is generally regarded as effective for managing venous congestion after fingertip replantation, few studies have systematically investigated standardized application protocols or their associated complications.

In this study, we compared two distinct postoperative protocols. The standardized approach used in Group B—prophylactic application two to three times daily from the early postoperative period—achieved a higher digit survival rate than the reactive, congestion-based protocol used in Group A, although the difference did not reach statistical significance. Early complications and reoperations were significantly less frequent in Group B. While leech therapy is traditionally applied after signs of congestion, our findings suggest that a proactive, standardized approach may improve clinical stability and outcomes. This protocol may serve as a practical guide for surgeons managing postoperative venous congestion in fingertip replantation.

### Outcomes of leech therapy following fingertip replantation

Maintaining stable surface bleeding after fingertip replantation is challenging.^29^ Our literature review showed higher survival with medicinal leech therapy—86 % (78/91 digits) in Tamai zone 1 and 84 % (38/45 digits) in zone 2^4^^,^^12^^,^^22^^,^^25^, ^26^, ^27^, ^28^—compared to earlier reports using surface bleeding alone, which reported 81.5 % (481/590) in zone 1 and 71 % (152/214) in zone 2.^29^ Leech therapy may thus offer a particular survival benefit in zone 2 injuries.

Few previous studies have assessed functional outcomes in replanted digits treated with leech therapy. In this study, both aesthetic and functional results were comparable to those reported after replantation with venous reconstruction.^1^^,^^2^^,^^20^ These data suggest that medicinal leech therapy can improve survival while yielding satisfactory clinical outcomes after fingertip replantation.

### Timing of leech application

The optimal timing for initiating medicinal leech therapy remains unclear. Our literature review demonstrated comparable survival rates between digits treated after the onset of venous congestion (76/83 digits; 92 %) and those treated prophylactically (15/16 digits; 94 %),^4^^,^^12^^,^^22^^,^^25^, ^26^, ^27^, ^28^ suggesting that reactive therapy can still be effective if started in the early stages of congestion. However, venous congestion is known to cause tissue injury earlier than arterial ischemia,^11^^,^^30^ and survival appears to be achievable only when therapy is initiated at the earliest stage of congestion.^31^

In our series, four cases of necrosis occurred in Group A, in which leeches were applied solely as salvage therapy following inadequate initial bleeding. During bloodletting, maceration often impairs accurate assessment of congestion, and delayed therapy based on overt clinical signs may worsen outcomes. We believe that these four necrotic cases in Group A resulted from delayed recognition of venous congestion, ultimately leading to necrosis. Several authors recommend initiating leech therapy at the first sign of congestion, as even brief delays can lead to arterial thrombosis.^12^^,^^13^ Taken together, these findings support the use of scheduled prophylactic leech application to standardize care, minimize tissue damage, and improve survival consistency.

### The importance of adjusting leech application frequency and its potential role in arterial insufficiency

Protocols for medicinal leech application vary, with reported intervals ranging from every 2 to 12 h or on a salvage basis.^4^^,^^10^^,^^22^^,^^32^ Post-detachment bleeding duration also varies (1–72 h).^11^ At our institution, an 8-h interval was adopted to balance leech availability, staff workload, patient burden, and bleeding risk.

In the early postoperative period, unstable digital circulation occasionally required more frequent application. In four digits, the initial leech remained attached for over an hour—previously linked to poor perfusion and outcomes.^27^ Prompt reapplication at <8-h intervals shortened subsequent attachment times, improved bleeding, and restored perfusion and Doppler signals. While primarily used to relieve venous congestion, leech secretions may also enhance local circulation and support mild arterial insufficiency.^11^^,^^13^ In one Group A digit, arterial vasospasm led to reoperation—possibly preventable with scheduled rather than salvage leeching.

In cases of unstable circulation, close monitoring of attachment time and bleeding is essential. If detachment is delayed or bleeding inadequate, immediate reapplication is warranted. Adjusting frequency accordingly may help maintain vascular stability.

### Duration of leech therapy

The optimal duration of leech therapy after fingertip replantation remains uncertain. Although 5–7 days is generally sufficient for establishing venous outflow,^10^^,^^18^^,^^33^ longer treatment may be required in elderly patients or those with extensive soft tissue damage.^18^ In our protocol, therapy typically ended on postoperative day 7. However, one Group B case developed partial necrosis after early discontinuation without proper monitoring—the pin-prick test was omitted. This underscores the need to avoid relying solely on a fixed duration. While Buntic et al.^12^ used fluorescein angiography, we routinely perform the pin-prick test to objectively assess perfusion. Confirming the absence of congestion through consistent evaluation is essential before ending therapy.

### Complications associated with leech therapy

Blood loss remains a major concern with leech therapy. Lee et al.^33^ reported increased transfusions, and Buntic et al.^12^ noted a 65 % transfusion rate with concurrent anticoagulation. In our series, only one anemic Group A patient required transfusion. In Group B, the prophylactic 8-h protocol resulted in a median hemoglobin drop of 2.5 g/dL (max 4.7 g/dL), without transfusion. Occasional excessive bleeding was controlled with light compression. Adjusting leech intervals, managing post-detachment bleeding, monitoring aPTT, and iron supplementation may help reduce transfusion risk.

Infection with leech-associated bacteria—particularly *Aeromonas hydrophila*—is another concern. While fluoroquinolones were once standard for prophylaxis, resistance has prompted the use of cefepime, carbapenems, or aminoglycosides.^13^^,^^34^^,^^35^ In our series, 10 of 25 digits (40 %) received only cefazolin, which is ineffective against *Aeromonas*. This pathogen can cause vasculitis, thrombosis, and tissue loss.^35^ Despite infection rates of 2.4–36.2 %, appropriate prophylaxis is often lacking; a UK study reported suitable antibiotics in only 19 % of cases.^35^ One Group B case developed late necrosis with *Aeromonas hydrophila* infection; proper antibiotics might have limited tissue loss. These findings underscore the need for effective antimicrobial coverage when using leeches after fingertip replantation.

### Limitations

This study has several limitations. First, it is a retrospective analysis with a small sample size, limiting statistical power. Second, procedures were performed by multiple surgeons, introducing potential variability in technique. Third, in cases where bloodletting was initiated before clear signs of venous congestion, it is possible that the digit might have survived without intervention. Additionally, Group B cases were more recent, so improved outcomes may reflect advancements in surgical skill rather than the protocol itself. Finally, the optimal leeching interval remains unclear and warrants further investigation.

## Conclusion

This study evaluated the use of leech therapy for venous congestion following fingertip replantation or revascularization in 25 digits, demonstrating an overall survival of 20 out of 25 digits (80 %) with favorable functional and aesthetic outcomes. Although the difference was not statistically significant, the group that received prophylactic leeching from the early postoperative period—prior to the onset of venous congestion (Group B: 12/13 digits; 92 %)—had a higher survival rate than the reactive-use group (Group A: 8/12 digits; 67 %). Early complications and reoperations were also more frequent in Group A.

These findings suggest that a standardized, proactive leeching protocol may improve survival and reduce complications, particularly in cases where venous anastomosis is not feasible.

## Declaration of AI and AI-assisted technologies in the writing process

During the preparation of this work the author used the large language model ChatGPT-4 (OpenAI, San Francisco, CA, USA) solely for language editing. After using this tool, the author reviewed and edited the content as needed and takes full responsibility for the content of the publication.

## Ethical approval

Approved by the institutional ethics committee (2023–017).

## Declaration of competing interest

None declared.
